# Book Reviews

**Published:** 1896-08

**Authors:** 


REPORT OF THE COMMISSIONER OF EDUCATION FOR THE YEAR 1892-93. Volumes I. and II. Washington: Government Printing Office. 1895.

It is a matter of surprise that so important a bureau of the government as that of education should delay the publication of its annual volume for so long a period of time. Many of the questions dealt with in it lose much of their force and effect in their presentation two or three years late. These remarks seem to us especially to apply to the chapter on medical education in the second volume. While there is much in it that will remain of value for reference, yet many things have happened since it was written and much progress has been made. Such a chapter from the highest educational authority in the country should be exhaustive and current. It should be made to contain everything of importance that has happened in the medical educational world for the previous year, and it should come to hand promptly. We hope the good work of methodising medical educational progress and recording it in annual governmental publication will continue and that it will be distributed to the profession with promptitude.

---

VOICE BUILDING AND TONE PLACING, Showing a New Method of Relieving Injured Vocal Cords by Tone Exercise. By H. HOLBROOK CURTIS, Ph. B., M. D., Fellow of the New York Academy of Medicine; Fellow of the American Laryngological, Rhinological and Otological Association. etc. Pp. 227. Illustrated. New York: D. Appleton & Co. 1896.

To most singers the importance of building the voice on a substantial basis and of preserving it with the utmost care is a matter of great moment, because upon it depend their fame and fortune. This is especially true of such artists as Jean and Edouard de Reszke and Mesdames Melba, Eames, Nordica and Patti. That they should have preserved their voices into middle life, maintaining them with the tone and freshness of youth, with an increasing power and richness of volume, indicates the careful training they have had and the discipline they maintain. While this book indicates many valuable points that pertain to voice building and tone placing, and even lays claim to a new method of relieving injured vocal cords by tone exercises, we still think that it has been written for the more particular purpose of advertising its author. His frequent reference by name to many of the noted singers of the period and his dedication of it to the most famous tenor of the times would seem to justify this conclusion. We would, however, commend it to the examination of all interested in the subject, for they will be able to find in it much of value.

---

TRANSACTIONS OF THE MEDICAL SOCIETY OF THE STATE OF NORTH CAROLINA. Forty-second annual meeting, held at Goldsboro, N. C., May 14, 15 and 16, 1895. Wilmington, N. C.: Legwin Brothers. 1895.

The medical society of the old North State always has an interesting meeting and publishes its work in accessible form. In this volume are to be found several valuable papers and much instructive discussion. In addition to the annual address of the president, this society has a fashion of appointing an annual essayist and an annual leader of debate on some definite subject. This year the essayist was Dr. R. H. Stancell, Jr., of Margaretsville, who chose for his subject Empiricism, which he handled in an able manner. The leader of the annual debate was Dr. A. A. Kent, of Lenoir, and his subject was the abuse of alcoholic stimulants in practice. This is an important question and was pointly dealt with. We think the space taken up by publishing the code of ethics might have been devoted to a better use.

---

TRANSACTIONS OF THE AMERICAN SURGICAL ASSOCIATON. Volume XIII. Edited by DEFOREST WILLARD, A. M., Ph. D., M. D., Recorder of the Association. Philadelphia : Printed for the Association and for sale by William J. Dornan. 1895.

The transactions of this association are always among the most important of the special societies. The president, Dr. Frederick S. Dennis, of New York, chose for the subject of his annual address, The etiology, diagnosis and treatment of malignant disease. There is no subject of greater import and none which demands more skill in diagnosis in its early manifestations. Dennis lays great stress on the influence of injury in the development of malignant growths, and we think his views are deserving of profound consideration. A series of papers in the discussion of the operative treatment of cancer in various localities is recorded in this book, which is easily the most comprehensive presentation of cancerous diseases that has appeared in recent literature. J. William White, of Philadelphia, presents a comprehensive paper on the results of double castration in hypertrophy of the prostate, which is manifestly a final determination of the subject to the present date. A discussion on anesthetics, participated in by Gay, of Boston ; Weir, of New York, and Deaver and Frese, of Philadelphia, is most timely and forceful. Park, of Buffalo, presents two papers—one on the consequences of hyperemia and the pathology of inflammation and suppuration, and another on the results of division in the neck of the pneumogastric and phrenic nerves. Mastin, of Mobile, offers an interesting clinical report of gunshot wound of the heart. Many other valuable papers go to make up one of the best volumes ever issued by this association, and it is easy to understand why every practical surgeon is anxious to possess it.

SYPHILIS IN THE MIDDLE AGES AND IN MODERN TIMES. By Dr. F. BURET, Paris, France. Translated from the French, with notes, by A. H. Ohmann-Dumesnil, M. D., Professor of Dermatology and Syphilology in the Marion Sims College of Medicine ; Consulting Dermatologist to the St. Louis City Hospital, to the St. Louis Female Hospital ; Physician for Cutaneous Diseases in the Alexian Brothers' Hospital ; Dermatologist to Pius Hospital, to the Rebekah Hospital, to the St. Louis Polyclinic and Emergency Hospital, etc., etc. Being Volumes II. and III. of Syphilis Today and Among the Ancients, complete in three volumes. Duodecimo, 300 pages. Extra cloth, $1.50 net. Philadelphia : The F. A. Davis Co., Publishers. 1895.

This interesting volume has been carefully written and faithfully translated. It is a classic history of a classic disease, and will prove valuable to every student of the subject. M. Buret is a scholarly and finished writer and Ohmann-Dumesnil has translated his idiom into smooth and fascinating English. Whoever would perfect his knowledge of the history of syphilis must assuredly possess this work.

---

TRANSACTIONS OF THE AMERICAN MICROSCOPICAL SOCIETY. Eighteenth annual meeting, held at Cornell University, Ithaca, N. Y., August 21, 22 and 23, 1895. Edited by WILLIAM C. KRAUSS, M. D., secretary, Buffalo, N. Y. Price, $2.50. Buffalo : The Wenborne-Sumner Co., Printers. 1896.

This handsomely printed and well-illustrated volume is a credit to the society whose proceedings it records. We could wish, however, that its binding was more substantial. A volume of 400 pages in paper is of short duration and of inconvenient reference. Those especially interested in preserving the book, no doubt, will incase it in cloth. Every student of microscopy will derive benefit from a study of this book, and, no doubt, will hasten to possess it.

